# Effects of Selective Serotonin Reuptake Inhibitors on Depression-Like Behavior in a Laser-Induced Shock Wave Model

**DOI:** 10.3389/fneur.2021.602038

**Published:** 2021-02-10

**Authors:** Soichiro Seno, Satoshi Tomura, Hiromi Miyazaki, Shunichi Sato, Daizoh Saitoh

**Affiliations:** ^1^Division of Traumatology, Research Institute, National Defense Medical College, Saitama, Japan; ^2^Division of Bioinformation and Therapeutic Systems, Research Institute, National Defense Medical College, Saitama, Japan

**Keywords:** mild blast traumatic brain injury, selective serotonin reuptake inhibitor, depression, neurogenesis, phosphorylated cAMP response element binding protein, brain-derived neurotrophic factor, laser-induced shock wave

## Abstract

Primary blast injury can result in depression-like behavior in the long-term. However, the effects of the selective serotonin reuptake inhibitor (SSRI) on the depression induced by mild blast traumatic brain injury (bTBI) in the long-term remain unclear. We generated a mouse model of mild bTBI using laser-induced shock wave (LISW) and administered an SSRI to mice by oral gavage for 14 days after LISW exposure. This study aimed to investigate the mechanisms of SSRI-mediated alleviation of depression-like behavior induced by mild bTBI. Animals were divided into three groups: sham, LISW-Vehicle, and LISW-SSRI. LISW was applied to the head of anesthetized mice at 0.5 J/cm^2^. Twenty-eight days after the LISW, mice in the LISW-SSRI group exhibited reduced depression-like behavior, a significant increase in the number of cells co-stained for 5-bromo-2'-deoxyuridine (Brd-U) and doublecortin (DCX) in the dentate gyrus (DG) as well as increased brain-derived neurotrophic factor (BDNF) and serotonin levels in the hippocampus compared to the sham and LISW-Vehicle groups. Additionally, levels of phosphorylated cAMP response element binding protein (pCREB) in the DG were significantly decreased in the LISW-Vehicle group compared to that in the sham group. Importantly, pCREB levels were not significantly different between LISW-SSRI and sham groups suggesting that SSRI treatment may limit the downregulation of pCREB induced by mild bTBI. In conclusion, recovery from depression-like behavior after mild bTBI may be mediated by hippocampal neurogenesis induced by increased BDNF and serotonin levels as well as the inhibition of pCREB downregulation in the hippocampus.

## Introduction

In recent years, the number of patients injured by bombs has increased globally (1). Many soldiers suffering from mild blast traumatic brain injury (mild bTBI) from the wars in Iraq and Afghanistan experience chronic mental disorders such as depression and cognitive impairments after returning to the United States (2–4). However, the mechanisms that lead to depression after mild bTBI remain unclear (2–5).

It is well established that neurogenesis occurs in the dentate gyrus (DG) and subventricular zone (SVZ) after traumatic brain injury (TBI). Neurogenesis persists for at least 1 month in the hippocampus and 2 weeks in the SVZ, although it can last for up to 1 year in humans (6). As previously reported, there is a negative relationship between depression and neurogenesis (7–12). In fact, novel theories regarding the mechanism of action of antidepressant drugs highlight activation of neurogenesis in the hippocampus. For example, stress can attenuate neurogenesis while the administration of antidepressant drugs activates neurogenesis in the hippocampal DG (7–11). Several previously published molecular studies may shed light on the relationship between depression and the hippocampal DG. Neurogenesis is also closely related with brain-derived neurotrophic factor (BDNF) (9, 11, 12). Regulation of BDNF expression involves phosphorylated cyclic adenosine monophosphate response element binding protein (pCREB) which acts as a gene transcription factor in cells. Chronic administration of antidepressant drugs increases pCREB, but the underlying mechanisms remain unclear (13, 14). Additionally, pCREB is thought to play an important role in hippocampal neurogenesis after TBI, but its precise mechanisms have not been fully elucidated (15–19).

Sato et al. developed a rat model of bTBI using laser-induced shock wave (LISW) (20). LISW is compact in size, easy to use and control, and has superior safety and versatility. Moreover, LISW enables the selection of specific energies or regions of application in animal models. Tomura et al. proposed a mild bTBI mouse model which recapitulates depression-like symptoms in the chronic phase after LISW exposure (21). However, the efficacy of early treatment with a selective serotonin reuptake inhibitor (SSRI) on depression-like behavior induced by mild bTBI in the chronic phase remain unclear. We hypothesized that any antidepressant-like effects would be closely related to neurogenesis, focusing especially on changes in BDNF, serotonin, and pCREB. Therefore, we administered an SSRI, which has been approved as the first-line drug for posttraumatic stress disorder (PTSD) and depression in the United States, in this mild bTBI mouse model to investigate the mechanisms of recovery from depression-like behaviors induced by mild bTBI (22).

## Materials and Methods

### Animals

**Figure 1:**
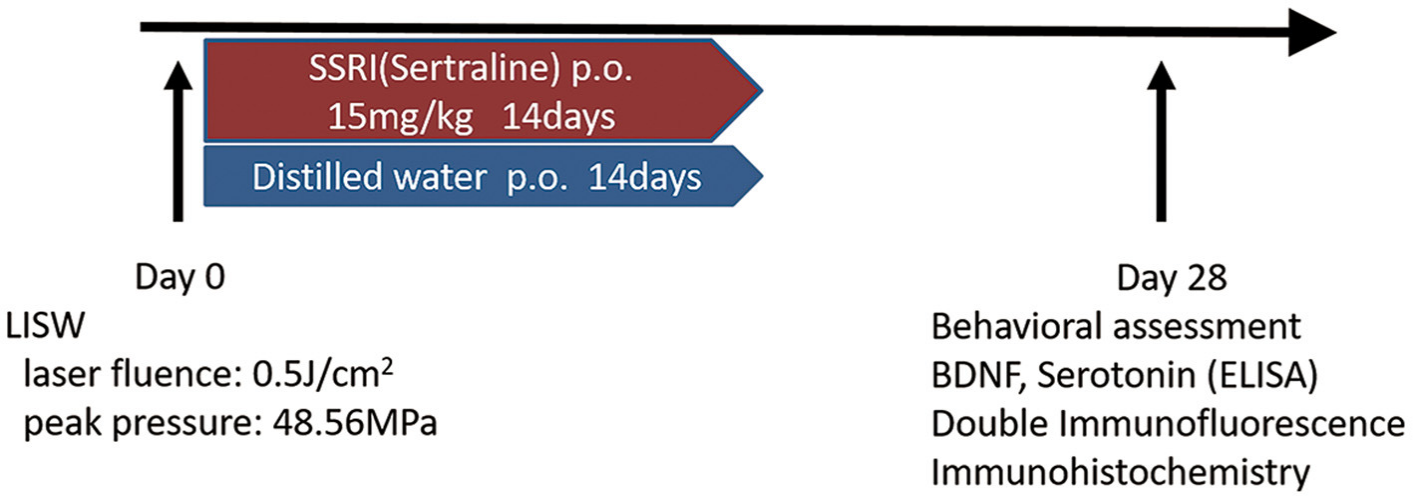
Schematic outline of the experimental schedule.

Male C57BL/6 mice (aged 8 weeks, weighing 22–25 g) were obtained from SLC Japan (Shizuoka, Japan). Mice were housed at 22–24°C with food and water available *ad libitum*. Animals were divided into three groups as follows; sham, LISW-Vehicle, and LISW-SSRI. Sertraline (FUJIFILM Wako Pure Chemical Corporation, Osaka, Japan) was selected as the SSRI and administered at 15 mg/kg to the SSRI group by oral gavage for 14 days after LISW exposure (23–26). The dose of sertraline was decided following a review of the literature (23, 24). Mice in the Vehicle and sham groups were administered distilled water instead of sertraline by oral gavage for 14 days. Sertraline or distilled water was administered to mice 24 h after LISW exposure on day 1. The sham-operated group underwent anesthesia and shaving of the head, but no LISW was applied (Figure 1). In this study, the mortality rate of the mice was zero. The study design was approved by the Ethics Committee of National Defense Medical College at the time of study initiation (approval number: 16010).

### LISW

**Figure 2:**
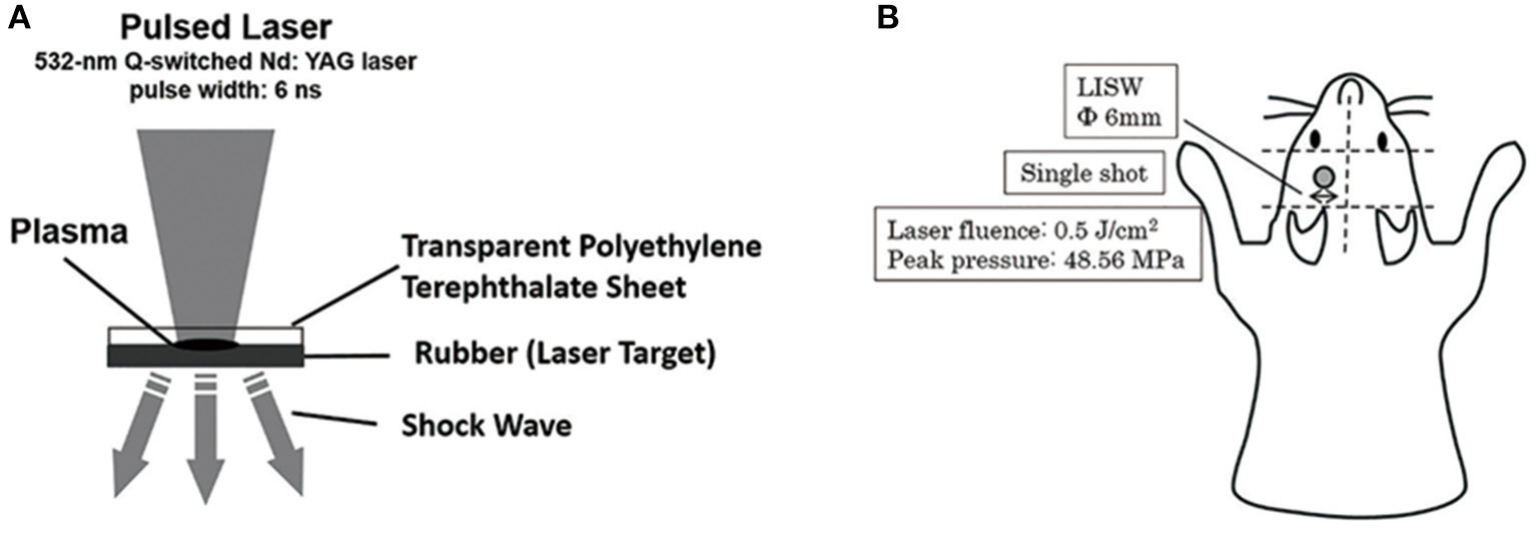
**(A)** Schema for the generation of laser-induced shock wave (LISW). A laser target comprising a light-absorbing material (0.5-mm-thick natural black rubber disk) on which an optically transparent material (1.0-mm-thick polyethylene terephthalate) was adhered with adhesive, was placed on the tissue. The laser pulse was absorbed by the black rubber to induce plasma and its expansion generated a shock wave (LISW). **(B)** This schema shows the region of LISW exposure. Single pulse by LISW was applied on the skin surface of the left parietal region. The laser target was 6-mm in diameter. Laser influence was 0.5 J/cm^2^, and peak pressure was 48.56 Mpa.

The second harmonics of a 532-nm Q-switched Nd: YAG laser (Brilliant b, Quantel, Les Ulis Cedex, France; pulse width, 6 ns) was used as described previously (20, 21, 27). A laser target comprising a light-absorbing material (0.5-mm-thick natural black rubber disk) on which an optically transparent material (1.0-mm-thick polyethylene terephthalate) was adhered with adhesive and placed on the tissue. The laser pulse was absorbed by the black rubber to induce plasma, and its expansion generated a shock wave (LISW) (27). The laser pulse was focused with a plano-convex lens to a 6-mm diameter spot on the target. The targets were carefully maintained using forceps when LISW was applied to the mice. Tomura et al. reported that LISW levels of 0.6 J/cm^2^ (peak pressure: 58.0 Mpa) and higher induced obvious skull bone fractures and brain surface hemorrhage (21). LISW levels of 0.5 J/cm^2^ (peak pressure: 48.6 Mpa) did not induce head fracture or hemorrhage. We therefore selected 0.5 J/cm^2^ as the threshold for inducing TBI to avoid traumatic injury to the head. In this study, LISW levels of 0.5 J/cm^2^ were used (21). A single LISW pulse was applied to the skin surface of the left parietal region (Figure 2).

### Behavioral Assessment

The tail suspension test and forced swimming test were performed as behavioral assessments at 28 days after LISW exposure. Both the tail suspension test and forced swimming test are well-established methods for assessing depression-like behavior (28–30). In the tail suspension test, mice were suspended by their tails using an elastic band attached to the tail by adhesive tape (approximately 1 cm from the tip of the tail), and the elastic band was hooked on a horizontal rod. Immobility time within a 6-min period was scored for each mouse after mice were suspended by a blinded observer (28, 29). In the forced swimming test, mice were placed in plastic cylinders (height, 30 cm; diameter, 20 cm) containing water (25 ± 1°C; depth, 15 cm). Immobility time within a 6-min period was scored for each mouse after they were placed in the water by a blinded observer (29).

The Y-maze test was also performed. The detailed protocols and results are presented in the Supplementary Material. Different animals were used in each behavioral experiment.

### ELISA Analysis

At 28 days after LISW exposure, mice were deeply anesthetized with a mix of ketamine and xylazine (ketamine: 100 mg/kg, xylazine: 10 mg/kg, intramuscular injection). Euthanasia was performed by cervical dislocation. The left total hippocampal area was rapidly dissected out and homogenized in RIPA buffer (Pierce® RIPA Lysis buffer; Pierce Biotechnology, Rockford, IL) containing protease inhibitors (The cOmplete® Roche, Penzberg, Germany). Samples were centrifuged at 4°C and 15,000 rpm for 10 min. The supernatants were collected for enzyme-linked immunosorbent assay (ELISA) analysis and stored at −80°C. The total protein concentration was determined using a BCA protein assay kit (Pierce™ BCA Protein Assay kit; Pierce Biotechnology).

BDNF protein levels in hippocampal homogenates were measured using the Mature BDNF Rapid™ ELISA kit (biosensis, Thebarton, South Australia) according to the manufacturer's instructions. Hippocampal homogenates (100 μL) were diluted with sample diluent and added to duplicate wells of plates pre-coated with an antibody against mature BDNF. After washing, an antibody against mature BDNF was added to the wells, and the plates were incubated for 30 min. After washing, a streptavidin-HRP conjugate was added to the wells, and the plates were incubated for 30 min. After washing, tetramethylbenzidine substrate was added to the wells for 8 min in the dark, then stop solution was added, and the plates were subsequently read on a plate reader at 450 nm. The calibration curve was plotted with the mean absorbance for the calibrator on the y-axis and concentration (0–500 pg/mL) of BDNF on the x-axis.

Serotonin levels in hippocampal homogenates were measured using the Abnova® Serotonin ELISA kit (Abnova, Taipei City, Taiwan) according to the manufacturer's manual. Hippocampal homogenates (100 μL) were diluted with sample diluent and added to duplicate wells of plates pre-coated with acylation reagent. Acylation buffer was added to the wells, and the plates were incubated for 30 min. Acylated samples were then added to duplicate wells in plates pre-coated with the serotonin antigen. A rabbit anti-serotonin antibody was added to the wells and plates ware incubated overnight at 4°C. After washing, goat anti-rabbit immunoglobulin conjugated with peroxidase was added to the wells, and the plates were incubated for 30 min. After washing, tetramethylbenzidine substrate was added to the wells for 30 min, stop solution was added, and the plates were read on a plate reader at 450 nm. The calibration curve was plotted using the mean absorbance for the calibrator on the y-axis against concentration (0–2.5 ng/mL) of serotonin on the x-axis. Each absorbance was read using SPECTRA max PLUS 384® (Molecular Devices Japan, Tokyo, Japan).

### Immunofluorescence Staining

At 28 days after LISW exposure, a separate group of mice were deeply anesthetized with a mixture of ketamine and xylazine (ketamine: 100 mg/kg, xylazine: 10 mg/kg, intramuscular injection) and transcardially perfused with normal saline followed by 4% paraformaldehyde. 5-Bromo-2'-deoxyuridine (Brd-U) staining was performed on paraffin sections using a Brd-U Labeling & Detection Kit II (Roche). Brd-U was injected intravenously (10 mL/kg) 24 h prior to euthanasia. Fifty micrometer-thick coronal sections were prepared from mouse brains, and slides were deparaffinized. For antigen retrieval, sections were autoclaved using a Decliaking Chamber™ (Biocare Medical, Pacheco, CA) with 10 mM citrate buffer (pH 6.0, Emergo Europe, Haag, Netherlands) for 15 min at 121°C. The sections were washed with tris-buffered saline (TBS) and incubated with blocking buffer (3% skim milk) for 30 min at 37°C. The sections were incubated overnight at 4°C with primary antibodies against DCX (1:800, Abcam, Cambridge, UK). The sections were washed with tris-buffered saline with tween (TBS-T) and TBS, and incubated for 60 min at 37°C with secondary antibodies (donkey anti-rabbit IgG antibody; 1:500, Abcam). The sections were washed with TBS-T and TBS, and incubated with blocking buffer (3% skim milk) for 30 min at 37°C. The sections were incubated for 30 min at 37°C with primary antibodies against Brd-U (Brd-U Labeling & Detection Kit II; Roche). The sections were washed with TBS-T and TBS and incubated for 30 min at 37°C with secondary antibodies (goat anti-mouse IgG antibody; 1:1,000, Abcam). The number of cells stained with both Brd-U and DCX in the left hippocampal DG was counted in a blinded manner using BZ-X710® (Keyence, Osaka, Japan). Six DG regions were selected from −2.06 to +2.54 mm anteroposterior to bregma in each mouse (21, 31).

### Immunohistochemical Staining

Coronal sections (50 μm-thick) were prepared for immunohistochemical analysis. Slides were deparaffinized and hydrated. For antigen retrieval, sections were autoclaved using a Decliaking Chamber™ (Biocare Medical) with 10 mM citrate buffer (pH 6.0, Emergo Europe) for 10 min at 110°C. Endogenous peroxidases were blocked with 3% hydrogen peroxide in methanol for 5 min. Further incubation with 10% goat serum (Nichirei Biosciences, Tokyo, Japan) was performed for 30 min at 37°C. The sections were incubated with primary antibodies against pCREB (1:5,000, Abcam) overnight at 4°C. The form of pCREB which we used was phosphorylated at Ser 133. The primary antibodies were diluted in 1% bovine serum albumin (Sigma-Aldrich Japan, Tokyo, Japan). The sections were washed with PBS and incubated with N-Histofine® Simple Stain Mouse MAX-PO (Nichirei Biosciences) for 30 min at room temperature. Peroxidase activity correlating to pCREB expression was visualized with diaminobenzidine (DAB; Histofine®, Nichirei Biosciences). One section per one animal was used for the analysis. For an evaluation of morphological changes, adjacent sections were counterstained with hematoxylin. The number of cells stained with DAB in the left hippocampal DG was counted in a blinded manner using BZ-X710® (Keyence). The cells stained with hematoxylin in the left hippocampal DG were counted for the total cells using BZ-X710®.

### Statistical Analysis

To estimate sample size, we referred to published data examining a mild bTBI model, wherein the immobility time in the forced swim test increased from 132.0 s in the sham group to 223.4 s in the LISW group (21). With an effect size of 1.36 and a standard deviation (SD) of 67.1, a sample size of 10 was estimated to provide 90% power in a two-sided test. All outcome measures were analyzed using one-way repeated measures analysis of variance, followed by *post hoc* analysis using Tukey's test. All data are expressed as mean ± standard deviation (± SD). Differences with *p* < 0.05 were considered statistically significant. The mice which could not swim were excluded in the forced swimming test. Other exclusion criteria did not exist. All statistical analyses were performed using SPSS ver. 24.0 for Windows (SPSS Inc., Chicago, IL).

## Results

### Behavioral Assessment

**Figure 3:**
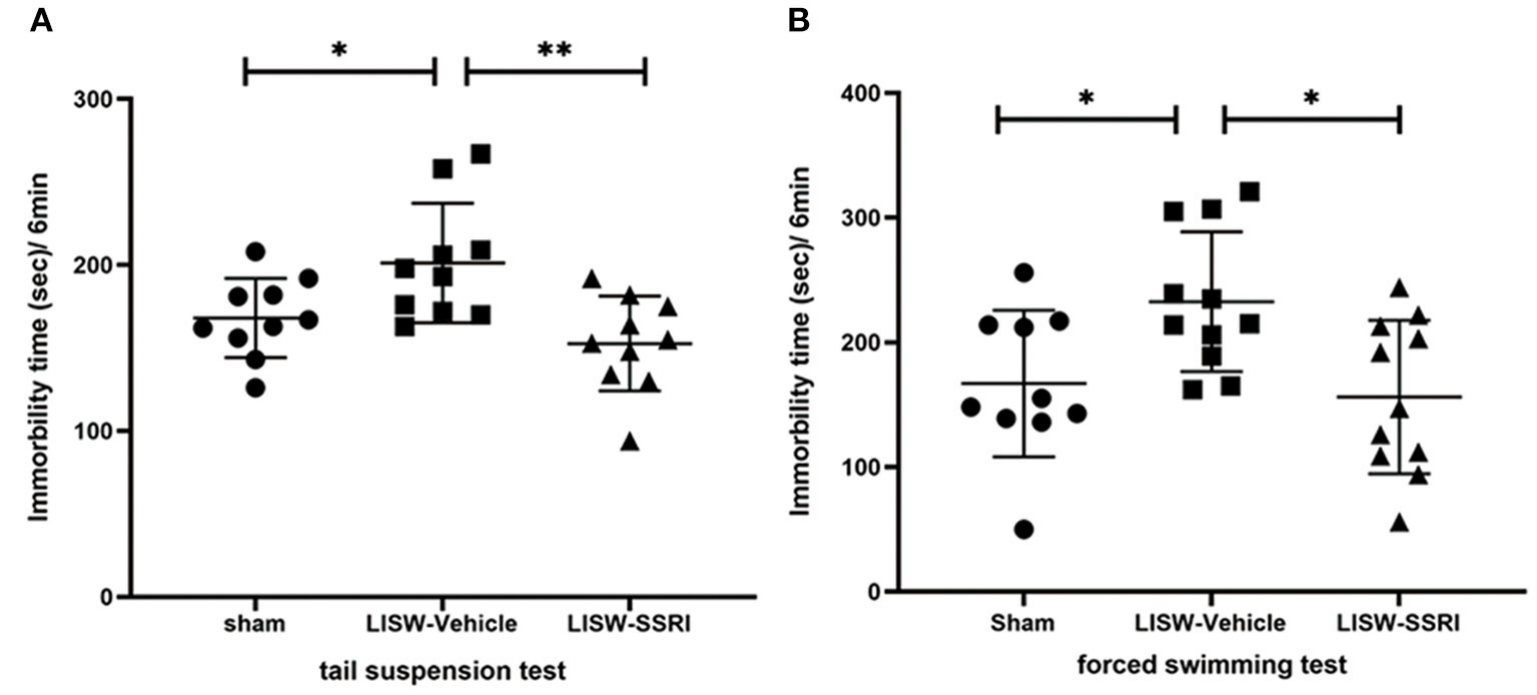
Results of behavioral assessments. In both the tail suspension test **(A)** and forced swimming test **(B)**, the immobility time in LISW-Vehicle group was significantly higher than that in the sham group. There was no significant difference between sham and LISW-SSRI groups; *n* = 10 **(A)**, *n* = 10–11 **(B)**, **p < 0.001, *p < 0.05.

In both the tail suspension and forced swimming tests, longer immobility time reflects greater depressive-like behavior (28–30). In the tail suspension test, immobility time was 168.0 ± 23.9, 201.2 ± 36.0, and 152.7 ± 28.6 s for sham, LISW-Vehicle, and LISW-SSRI groups, respectively, at 28 days after LISW exposure (*n* = 10). Immobility time was significantly longer in the LISW-Vehicle group than in the sham group (F_2, 27_ = 6.876, *p* < 0.05). No significant differences between sham and LISW-SSRI groups were observed (Figure 3A).

In the forced swimming test, the immobility time was 167.0 ± 58.9, 232.5 ± 56.1, and 156.2 ± 61.5 s for sham, LISW-Vehicle, and LISW-SSRI, respectively, at 28 days after LISW exposure (*n* = 10–11). Immobility time was significantly longer in the LISW-Vehicle group than in the sham group (F_2, 29_ = 5.373, *p* < 0.05). No significant differences between sham and LISW-SSRI groups were observed (Figure 3B).

### BDNF and Serotonin in the Left Hippocampal DG

**Figure 4:**
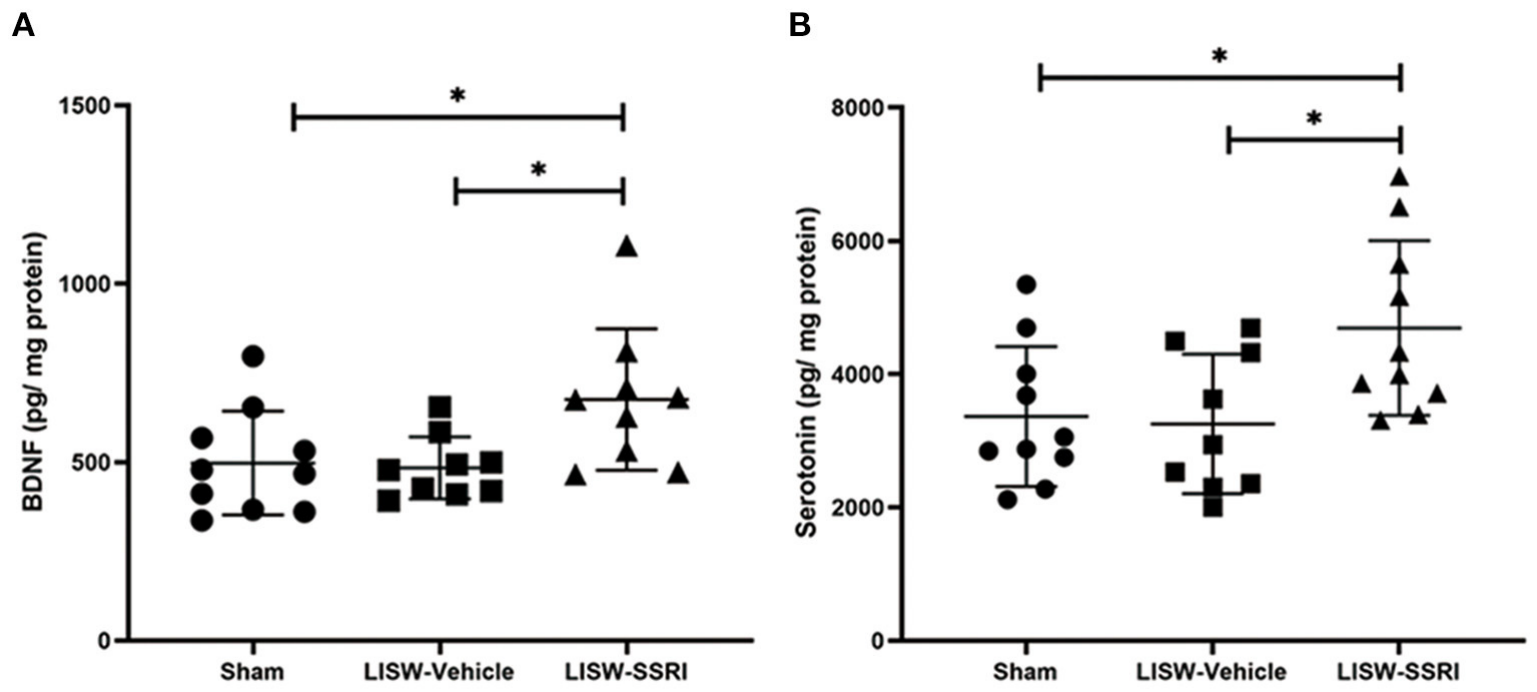
Graphical presentation of BDNF and serotonin levels in the left hippocampal DG. **(A)** BDNF protein levels in the left hippocampus in the LISW-SSRI group were significantly higher than those in the sham and LISW-Vehicle groups. There was no significant difference between sham and LISW-Vehicle groups. **(B)** Serotonin levels in the left hippocampus of the LISW-SSRI group were significantly higher than those of the sham and LISW-Vehicle groups. No significant differences between sham and LISW-Vehicle groups were noted; *n* = 9–10 for each test, *p < 0.05.

At 28 days after LISW exposure, BDNF protein levels in the left hippocampal DG was 497.6 ± 145.4, 484.2 ± 87.2, and 675.8 ± 198.1 pg/mg protein in sham, LISW-Vehicle, and LISW-SSRI groups, respectively (*n* = 9–10). BDNF protein levels in the LISW-SSRI group were significantly higher than those in the sham and LISW-Vehicle groups (F_2, 25_ = 4.620, *p* < 0.05). No significant differences between sham and LISW-Vehicle groups were noted (Figure 4A).

At 28 days after LISW exposure, serotonin levels in the left hippocampal DG were 3,364.8 ± 1,050.2, 3,253.2 ± 1,047.6, and 4,692.2 ± 1,311.7 pg/mg protein in sham, LISW-Vehicle, and LISW-SSRI groups, respectively (*n* = 9–10). Serotonin levels in the LISW-SSRI group were significantly higher than those in the sham and LISW-Vehicle groups (F_2, 26_ = 4.768, *p* < 0.05). No significant differences between sham and LISW-Vehicle groups were noted (Figure 4B).

### Co-expression of Brd-U and DCX in the Left Hippocampal DG

Brd-U is incorporated into newly synthesized DNA during the S period. DNA containing Brd-U can be detected using an anti-Brd-U antibody, which enables the detection of proliferating cells that replicate DNA (32). Doublecortin (DCX) is expressed by differentiated neurons. As such, co-staining with both Brd-U and DCX enables the analysis of proliferated neurons, indicating neurogenesis (15, 33).

**Figure 5:**
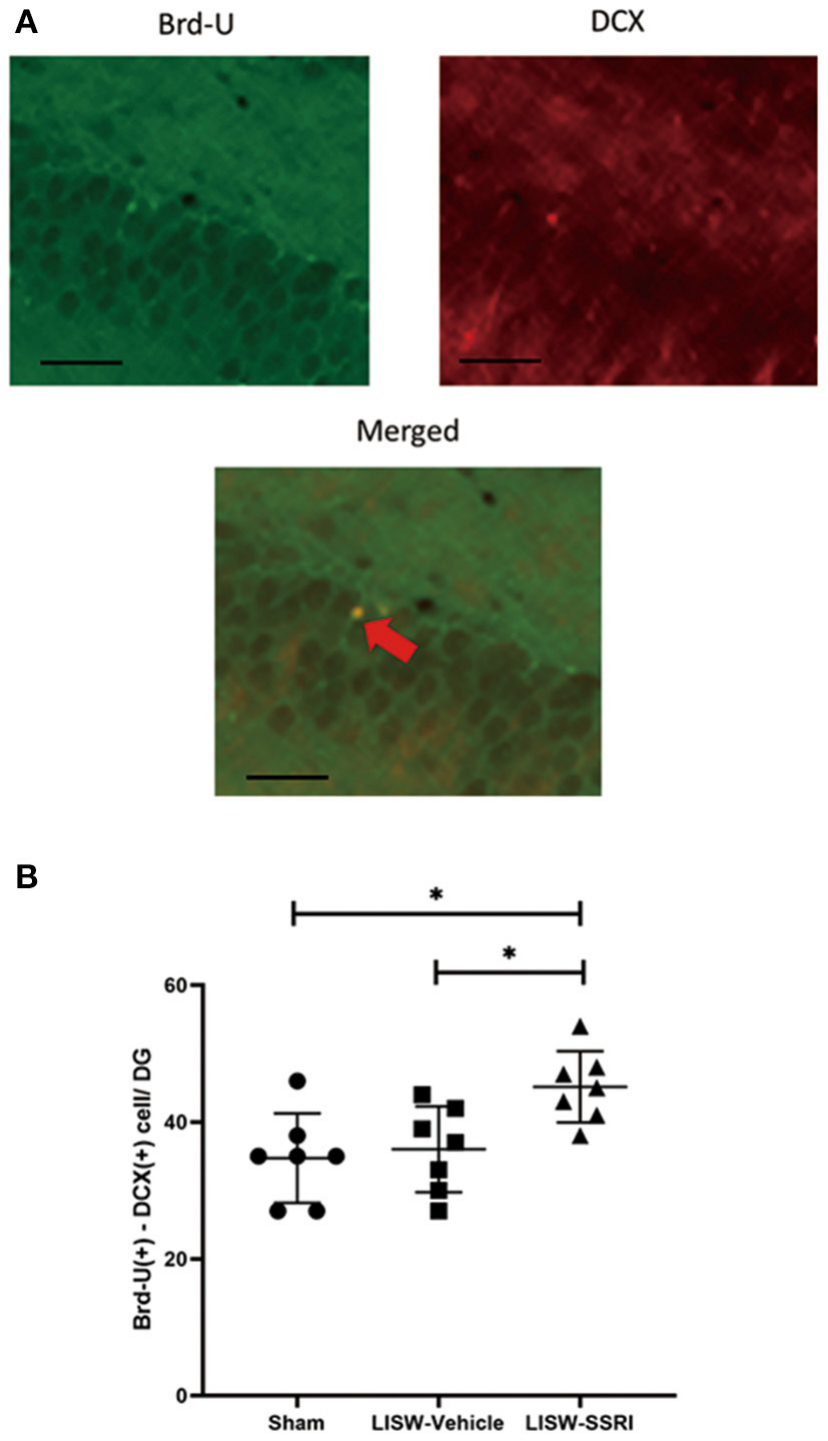
Co-expression of Brd-U and DCX in the left hippocampal DG. **(A)** For immunofluorescence staining, cells stained with Brd-U are colored in green and those stained with DCX are colored in red, Double-labeled cells stained with both Brd-U and DCX appear yellow. **(B)** In the left hippocampal DG, the number of cells stained with both Brd-U and DCX in the LISW-SSRI group was significantly higher than that in the sham and LISW-Vehicle groups; scale bars = 25 μm, *n* = 7, *p < 0.05.

At 28 days after LISW exposure, we compared cells stained with both Brd-U and DCX in the left hippocampal DG. For immunofluorescence staining, cells stained for Brd-U are indicated in green and those stained for DCX are indicated in red. Double-labeled cells appear yellow (Figure 5A).

The number of cells stained with both Brd-U and DCX was 34.7 ± 6.6, 36.0 ± 6.3, and 45.1 ± 5.2 for sham, LISW-Vehicle, and LISW-SSRI, groups, respectively (*n* = 7). The number of cells stained with both Brd-U and DCX in the LISW-SSRI group was significantly higher than that in sham and LISW-Vehicle groups (F_2, 18_ = 6.208, *p* < 0.05) (Figure 5B).

### Ratio of pCREB-Positive Cell Counts in the Left Hippocampal DG

**Figure 6:**
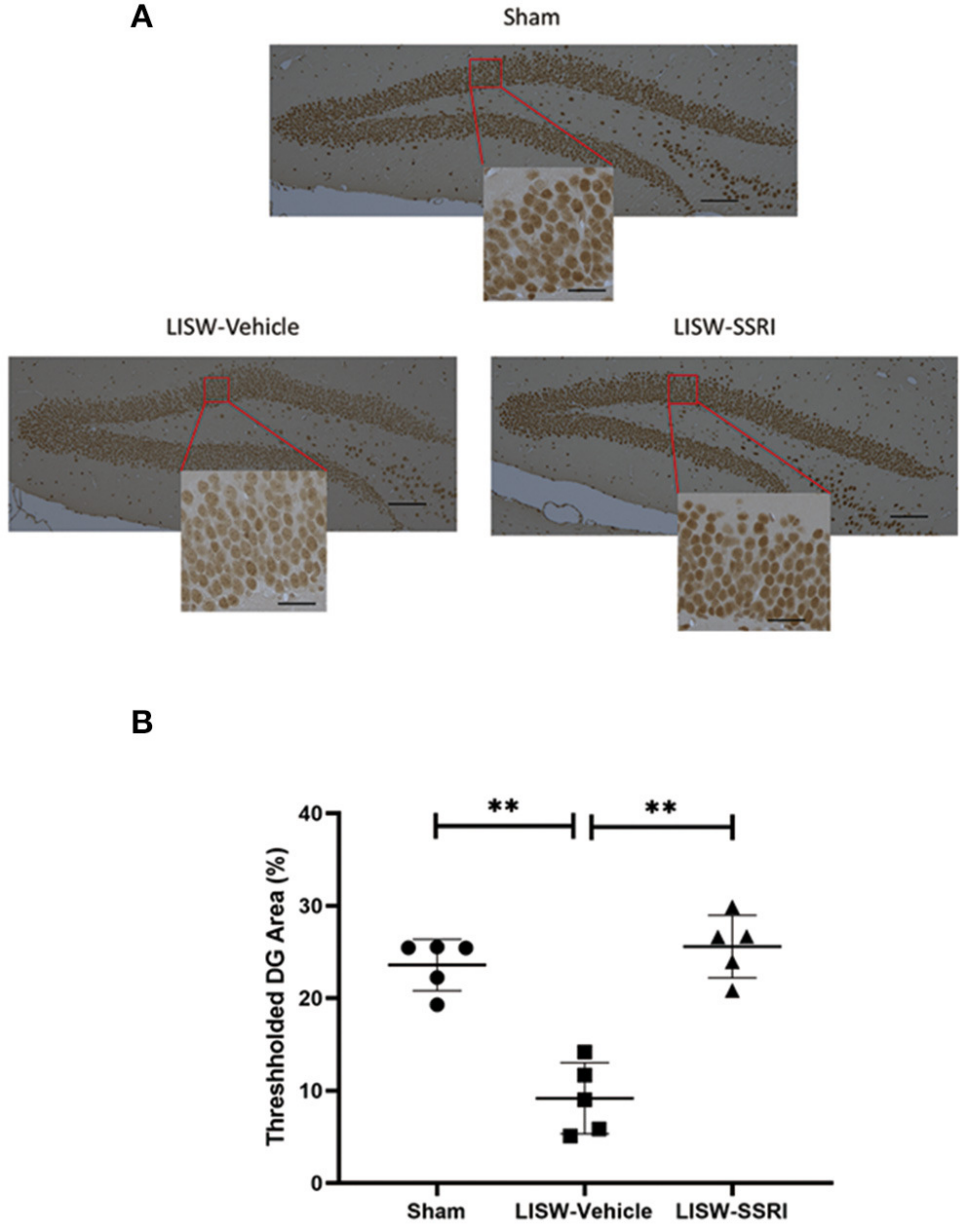
Ratio of pCREB-positive cell counts in the left hippocampal DG. **(A)** The ratio of pCREB-positive cells (number of positive cells stained with pCREB antibody / total number of cells) in the left hippocampal DG was calculated in a blinded manner using BZ-X710® (Keyence). **(B)** The ratio of positive cells in the LISW-Vehicle group was significantly lower than that in the sham and LISW-SSRI groups. There was no significant difference between sham and LISW-SSRI groups; scale bars = 100 and 25 μm, *n* = 5, **p < 0.001.

At 28 days after LISW exposure, we compared the number of cells positive for pCREB based on DAB staining in the left hippocampal DG. We calculated the ratio (number of cells with positive staining using the pCREB antibody/total number of cells) in a blinded manner using BZ-X710® (Keyence). We compared the ratio of positive cells in the left hippocampal DG (Figure 6A), which was 23.6 ± 2.8%, 9.2 ± 3.8%, and 25.6 ± 3.4% for sham, LISW-Vehicle, and LISW-SSRI, groups, respectively (*n* = 5). The ratio of positive cells was significantly lower in the LISW-Vehicle group than in the sham and LISW-SSRI groups (F_2, 12_ = 35.457, *p* < 0.001). No significant differences between the sham and LISW-SSRI groups were observed (Figure 6B).

## Discussion

In this study, we examined the effects of SSRI administration for 2 weeks following TBI on behavior at 1 month post injury in a mouse model of mild bTBI using LISW. Our data showed that administration of an antidepressant drug alleviated depression-like behavior at 28 days after LISW exposure. When given prior to the development of symptoms, SSRIs may be an effective therapeutic strategy for prevention of depression during the chronic phase of a mild TBI. SSRIs increase the extracellular levels of the neurotransmitter serotonin by limiting reuptake into presynaptic cells, thereby increasing the level of serotonin in the synaptic cleft. The half-life of sertraline is 22–24 h. The theory of the monoamine hypothesis is that the depletion of serotonin and other monoamines is the pathophysiology underling depression (34). However, the monoamine hypothesis has several contradictions. For example, the elevation of monoamines in the synaptic cleft occurs within a short period of time after the administration of an antidepressant drug. Despite this rapid elevation in monoamines from the first administration, typical antidepressants take weeks to months of chronic use to exert their therapeutic effects (34, 35). In this study, the serotonin levels in the LISW-Vehicle group were the same as those of the sham group at 28 days after LISW exposure. Our results contradict the monoamine hypothesis. Some literature has reported the utility of sertraline for prophylaxis following TBI (36, 37). In this study, sertraline was administered to the mice for 14 days, not for 28 days. Van Dyke et al. reported that the effects of elevated serotonin accumulate slowly *in vivo* and may account for the delay in relief of depression (38). Our sertraline treatment might partially prevent the development of depression and may have mechanisms other than that of the monoamine hypothesis.

In recent years, increasing focus has been placed on the role of neurogenesis in anti-depressant mechanisms. Therefore, we focused on neurogenesis as the potential mechanism of recovery from depression-like behavior induced by mild bTBI. Neurogenesis is the physiological phenomenon by which new neurons differentiate from neural stem cells or precursor cells. It occurs in embryonic and fetal periods. Neurogenesis plays an important role in the formation and development of the brain. New neurons decrease as an individual develops, but neurogenesis persists in the hippocampus and SVZ even after maturation (11). We observed an increase in the number of cells stained with both Brd-U and DCX in the hippocampal DG of the LISW-SSRI group 28 days after LISW exposure. This suggests that neurogenesis in the DG was activated by early treatment with an SSRI. This neurogenesis at 28 days might be strongly related to the SSRI treatment, not due to mild bTBI. As described in the Introduction, exposure to stress reduces neurogenesis, but the administration of antidepressant drugs activates neurogenesis in the DG (7–10). For example, Malberg et al. reported that chronic administration of an SSRI activated neurogenesis in the hippocampal DG (8). Therefore, the antidepressant-like effects in the SSRI-LISW group observed in this study may have been influenced by neurogenesis induced by SSRI administration.

The detailed mechanisms underscoring neurogenesis are not fully known, but our understanding of the relationship between neurogenesis and signaling molecules is growing. In this study, we focused on BDNF, serotonin, and pCREB, which are factors related to neurogenesis in the hippocampus. BDNF is a neurotrophic factor belonging to the neurotrophic family, which also includes nerve growth factor. It acts to maintain neural cells, promote neurite outgrowth, and regulate neurotransmitter synthesis. BDNF and serotonin levels were increased in the hippocampus of the SSRI-LISW group. This is likely due to the fact that SSRI administration limits the reuptake of serotonin in the synaptic cleft. SSRIs also contribute to elevation of BDNF in the synaptic cleft, and BDNF can interact with serotonin in the synaptic cleft (11, 39). In short, BDNF and serotonin interact each elevating the level of the other in the synaptic cleft (39). In this study, serotonin may have interacted with BDNF in the hippocampus after SSRI administration, which could have induced an activation of neurogenesis in the hippocampal DG.

The pCREB is associated with BDNF gene expression in the cytoplasm via various pathways. The pCREB can be activated after phosphorylation by several signaling molecules via the BDNF – pCREB pathway. Under TBI conditions, the basal levels of pCREB decrease over several months post-trauma (40). Wu et al. reported that pCREB was downregulated in the hippocampus after TBI (15). Similarly, we found that pCREB was significantly lower in the hippocampal DG of the LISW-Vehicle group compared to the sham group. This result suggests that LISW exposure could influence the downregulation of pCREB. Conversely, there was no significant difference between sham and LISW-SSRI groups. Therefore, SSRI administration may have limited the downregulation of pCREB induced by mild bTBI. SSRI administration promotes the elevation of BDNF and serotonin in the synaptic cleft. The elevation of BDNF and serotonin could also play an important role in the downregulation of pCREB. However, the relationship between BDNF or serotonin and downregulation of pCREB is unknown thus requiring further examination in the future.

In this study, we used LISW to investigate the effects of early SSRI treatment on bTBI-induced depression-like symptomology 28 days later. The shock tube or open field blast models are often used in studies on blast injuries (41, 42). However, compared to conventional instruments, LISW has higher controllability of various parameters such as pressure, impulse, frequency, and regions (20, 27). While we applied a single pulse by LISW on the skin surface of the left parietal region in mice, it is important to note that alterations in various conditions such as species, side, frequency, or region may provide even more insight into the mechanisms of bTBI. For instance, rats could be used instead of mice; the LISW could have performed on the right side, cerebellum, or frontal lobe; or a more severe bTBI model could have been used instead of a mild bTBI model. Therefore, future experiments using different parameters or bTBI models should be compared to those from this model in an attempt to better understand the mechanisms involved. Moreover, future studies should use another control group where shams are treated with the SSRI, or use female mice, or different strains instead of male C57BL/6 mice.

We chose to study the effects of an SSRI in this study given that they are often used as the first-line pharmacological treatment for PTSD and depression in the United States (22). However, it will be important to investigate other pharmacological treatments with different mechanisms of action other than SSRI's using our model in the future. This could help shed further light on the mechanisms involved in the successful recovery from bTBI-induced depression. It is also important to note that other factors such as tropomyosin receptor kinase B (TrkB) in the BDNF - pCREB pathway have been previously associated with depression (43). Therefore, novel mechanisms may also be identified by investigating the relationship between these other factors and neurogenesis. Further, SSRIs have been shown to play an important role in transporting other monoamines such as dopamine or noradrenaline, making it important to examine other monoamine systems in the future (44).

This study has several limitations. First, we only analyzed a single time point 28 days after LISW exposure. This time point was selected because many soldiers who suffered from mild bTBI experienced depression during the chronic phase, not the acute phase (2–4). Nevertheless, studies geared at examining changes in neurogenesis during acute phase are still warranted. Hence, we should check other time points, such as 7 or 14 days. It will be interesting to compare the findings at 28 days and other time points. Another limitation is that we only examined the ipsilateral side of the hippocampus after LISW. We selected the hippocampus because it is one of the most well established sites of neurogenesis in TBI (6). Sahay et al. revealed that the heterogeneity of depression indicates that its origin may lie in dysfunction of multiple brain regions (45). SSRI treatment also impacts the entire brain. This model is limited in that although bTBI affects all brain regions, the exposure disproportionately affects the hippocampus. Hence the effect of exposure to other brain regions is not included in this study. It is possible that exposure to all regions may have an effect, but we expect that to be limited since anxiety and depression predominantly occur due to hippocampal dysfunction. Moreover, we only examined depression, and other deficits such as anxiety should also be examined. The study of various deficits in mice may clarify the mechanism of SSRIs.

In conclusion, early administration of an SSRI produced an elevation in BDNF and serotonin and attenuated pCREB downregulation in the hippocampus after LISW exposure. These changes may underlie the increase in neurogenesis observed after SSRI treatment in the hippocampal DG and ultimately lead to the amelioration of depression-like behavior induced by mild bTBI.

## Data Availability Statement

The raw data supporting the conclusions of this article will be made available by the authors, without undue reservation.

## Ethics Statement

The animal study was reviewed and approved by the Ethics Committee of National Defense Medical College (approval number: 16010).

## Author Contributions

SSe was the primary investigator of this study and was thus responsible for all the study processes. ST contributed to study design, data interpretation, and revision of the manuscript. HM contributed to data collection and data interpretation. SSa contributed to resources and study design. DS contributed to study design, statistical analysis, data interpretation, revision of the manuscript, and provided final approval to submit the manuscript for publication. All authors contributed to the article and approved the submitted version.

## Conflict of Interest

The authors declare that the research was conducted in the absence of any commercial or financial relationships that could be construed as a potential conflict of interest.
