# Supplementary material for: Effects of Selective Serotonin Reuptake Inhibitors on Depression-Like Behavior in a Laser-Induced Shock Wave Model
Source: Front Neurol. 2021 Feb 10;12:602038. doi: 10.3389/fneur.2021.602038 (PMC7902879; doi:10.3389/fneur.2021.602038)
Supplement: Supplementary file 1 [file Data_Sheet_1.PDF]

Effects of Selective Serotonin Reuptake Inhibitors on Depression-like Behavior in a Laser-induced Shock Wave Model

(Running title: SSRI Treatment in LISW model)

Soichiro Seno, M.D., Ph.D.[*1]*; Satoshi Tomura, M.D., Ph.D.*[1]*; Hiromi Miyazaki, Ph.D.*[1]*; Shunichi Sato, Ph.D.*[2]*; Daizoh Saitoh, M.D., Ph.D.*[1]

[1]*Division of Traumatology, Research Institute, National Defense Medical College, Saitama, Japan*

[2]*Division of Bioinformation and Therapeutic Systems, Research Institute,*

National Defense Medical College, Saitama, Japan

ADDRESS FOR CORRESPONDENCE

Soichiro Seno

Division of Traumatology, Research Institute, National Defense Medical College,

3-2 Namiki, Tokorozawa, Saitama, 359-8513, Japan

Phone: +81-4-2995-1511

Fax: +81-4-2996-5221

E-mail: soichiro.seno.scholar@gmail.com

Methods

Y-maze test

We examined short-term memory at 28 days after mild bTBI using the Y-Maze Spontaneous Alternation Test, as previously described (1,2). Our symmetrical Y-maze was made of acrylic material and consisted of three arms (25 × 5 cm) separated at 120° angles from each other with 15-cm-high transparent walls. Each mouse was placed in the center of the Y-maze and was allowed to freely explore the maze for 8 min. Typically, mice prefer to explore a new branch rather than return to the previously visited one. This is referred to as an alternation. The sequence and total number of arms entered were recorded. The percentage of alternations was calculated by taking the number of triads containing entries into all three arms and dividing by the maximum possible number of alternations (total number of arm entries - 2) × 100.

Results

In the Y-maze test, the discrimination index was 0.628 ± 0.102, 0.525 ± 0.089, and 0.588 ± 0.108 for the sham, LISW-Vehicle, and LISW-SSRI groups, respectively, at 28 days after LISW exposure (n = 10). No significant differences among the sham, LISW-Vehicle, and LISW-SSRI groups were observed ($F_{2,27} = 2.709$, $p = 0.085$).

REFERENCES

1. Tomura S, Seno S, Kawauchi S, Miyazaki H, Sato S, Kobayashi Y, et al. A novel mouse model of mild traumatic brain injury using laser-induced shock waves. *Neurosci Lett.* (2020);721:134827. doi: 10.1016/j.neulet.2020.134827
2. Miyazaki H, Miyawaki H, Satoh Y, Saiki T, Kawauchi S, Sato S, et al. Thoracic shock wave injury causes behavioral abnormalities in mice. *Acta Neurochir (Wien).* (2015);157(12):2111-20; discussion 20. doi: 10.1007/s00701-015-2613-3

Supplementary Figure

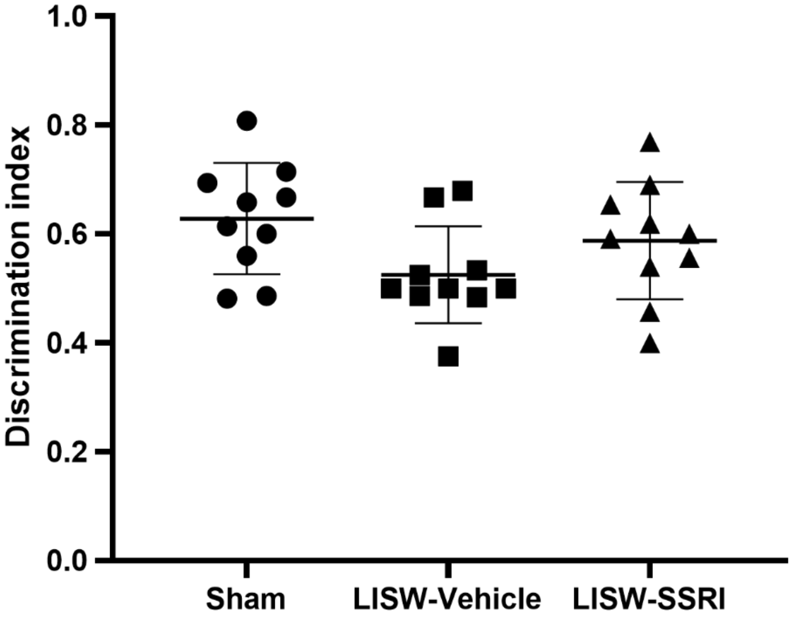

Supplementary Table 1

tail suspension test (sec)

Sham	LISW-Vehicle	LISW-SSRI
162	258	164
167	209	148
192	170	155
181	172	153
126	267	192
163	176	130
156	198	175
182	163	182
208	193	94
143	206	134

Supplementary Table 2

forced swimming test (sec)

Sham	LISW-Vehicle	LISW-SSRI
50	307	192
136	214	147
217	206	213
148	235	244
139	305	56
155	321	222
256	189	109
212	215	112
143	165	203
214	162	94
	239	126

Supplementary Table 3

Y-maze		
Sham	LISW-Vehicle	LISW-SSRI
0.808	0.500	0.540
0.614	0.500	0.591
0.486	0.679	0.769
0.658	0.484	0.400
0.600	0.500	0.619
0.560	0.375	0.556
0.481	0.667	0.690
0.694	0.533	0.600
0.667	0.525	0.654
0.714	0.486	0.457

Supplementary Table 4

BDNF (pg/ mg protein)		
Sham	LISW-Vehicle	LISW-SSRI
532.1212121	499.7362405	810.8108108
367.1369295	478.0298385	707.0847851
412.2040073	654.1807637	675.5247879
653.8892625	583.9562443	533.1209123
361.0928242	391.8830102	466.3888386
336.9768894	409.4336314	681.3214108
479.2888563	493.9818631	1107.946027
568.0272109	427.0800873	627.9946761
797.3303671	419.2414431	472.0177991
467.8138942		

Supplementary Table 5

Serotonin (pg/ mg protein)		
Sham	LISW-Vehicle	LISW-SSRI
2873.246753	2532.618252	3716.604667
3053.941909	4322.092786	4332.345313
2752.823315	3628.081199	3867.574810
2117.686617	2302.096627	6512.326010
3681.533904	2360.170884	3313.100199
5345.096814	2940.031562	3992.051664
2845.857771	2004.946414	6972.263868
4697.035957	4693.829854	3397.443890
4004.004449	4495.451237	5648.624667
2276.768642		5170.118090

Supplementary Table 6

Brd-U(+)-DCX(+) cell/ DG		
Sham	LISW-Vehicle	LISW-SSRI
27	42	45
35	44	48
35	39	43
35	27	38
38	37	41
27	30	47
46	33	54

Supplementary Table 7

pCREB

Sham			LISW-Vehicle			LISW-SSRI		
positive	total	ratio (%)	positive	total	ratio (%)	positive	total	ratio (%)
15187	59728	25.4269354	2571	21993	11.6900832	11080	46267	23.9479543
10261	46163	22.2277582	4729	33346	14.1816110	14427	48281	29.8813198
21528	84582	25.4522239	2701	29961	9.0150529	12598	47323	26.6213047
11499	59575	19.3017205	1659	32483	5.1072869	9854	47296	20.8347429
13357	52318	25.5304102	1308	22366	5.8481624	17547	65819	26.6594752
